# The Functional Role and Regulatory Mechanism of FTO m^6^A RNA Demethylase in Human Uterine Leiomyosarcoma

**DOI:** 10.3390/ijms24097957

**Published:** 2023-04-27

**Authors:** Qiwei Yang, Ayman Al-Hendy

**Affiliations:** Department of Obstetrics and Gynecology, University of Chicago, Chicago, IL 60637, USA

**Keywords:** uterine leiomyosarcoma, RNA methylation, demethylase, DAC51, transcriptome analysis, apoptosis, cell cycle, histone modifications, transcription factors, miRNAs

## Abstract

Uterine leiomyosarcoma (uLMS) is the most frequent subtype of uterine sarcoma that presents a poor prognosis and high rates of recurrence and metastasis. The origin and molecular mechanism underlying and driving its clinical and biological behavior remain largely unknown. Recently, we and others have revealed the role of microRNAs, DNA methylation, and histone modifications in contributing to the pathogenesis of uLMS. However, the connection between reversible m^6^A RNA methylation and uLMS pathogenesis remains unclear. In this study, we assessed the role and mechanism of FTO m^6^A RNA demethylase in the pathogenesis of uLMS. Immunohistochemistry analysis revealed that the levels of RNA demethylases FTO and ALKBH5 were aberrantly upregulated in uLMS tissues compared to adjacent myometrium with a significant change by histochemical scoring assessment (*p* < 0.01). Furthermore, the inhibition of FTO demethylase with its small, potent inhibitor (Dac51) significantly decreased the uLMS proliferation dose-dependently via cell cycle arrest. Notably, RNA-seq analysis revealed that the inhibition of FTO with Dac51 exhibited a significant decrease in cell-cycle-related genes, including several CDK members, and a significant increase in the expression of *CDKN1A*, which correlated with a Dac51-exerted inhibitory effect on cell proliferation. Moreover, Dac51 treatment allowed the rewiring of several critical pathways, including TNFα signaling, KRAS signaling, inflammation response, G_2_M checkpoint, and C-Myc signaling, among others, leading to the suppression of the uLMS phenotype. Moreover, transcription factor (TF) analyses suggested that epitranscriptional alterations by Dac51 may alter the cell cycle-related gene expression via TF-driven pathways and epigenetic networks in uLMS cells. This intersection of RNA methylation and other epigenetic controls and pathways provides a framework to better understand uterine diseases, particularly uLMS pathogenesis with a dysregulation of RNA methylation machinery. Therefore, targeting the vulnerable epitranscriptome may provide an additional regulatory layer for a promising and novel strategy for treating patients with this aggressive uterine cancer.

## 1. Introduction

Uterine leiomyosarcoma (uLMS) is a rare and aggressive uterine cancer, representing 1–2% of all uterine malignancies [1]. The annual incidence of uLMS is approximately 0.8 per 100,000 women. The five-year survival for all patients is between 25 and 76%, with survival for women with metastatic disease at the initial diagnosis approaching only 10–15% [2]. Although irrespective of treatment, the uLMS is characterized by poor prognosis [3], and presently, uLMS patients exhibit resistance to currently available therapies, as evidenced by high recurrence and progression rates [4]. In addition, the origin and mechanism underlying and driving its clinical and biological behavior remain unclear [5,6].

Among different chemical modifications in RNA, N6-methyladenosine (m^6^A), methylated at the N6 position of adenosine, accounts for the most pervasive, abundant, and conserved internal transcriptional modification within eukaryotic mRNAs, microRNAs, and long non-coding RNAs, and has emerged as a widespread regulatory mechanism that controls gene expression in diverse physiological processes [7,8]. m^6^A modification can stabilize RNAs and modulate their localization, transport, and post-translational regulation. The process of RNA methylation is catalyzed by methyltransferases (also known as “writers”), including methyltransferase-like 3 (METTL3), METTL14, and their cofactors such as Wilms’ tumor 1-associated protein (WTAP) and RNA-binding motif protein 15 (RBM15) [9]. The m^6^A modification can be reversed by demethylases (also known as “erasers”) such as fat mass and obesity-associated protein (FTO) and AlkB homologue 5 (ALKBH5) [10]. The m^6^A can influence post-transcriptional gene expression during transcription through specific recognition by m^6^A-binding proteins (also known as “readers”), such as YTH domain-containing proteins and IGF2BP1-3 [11,12]. Given the critical oncogenic role of RNA demethylases in many types of cancers, the study of FTO and its relevant products has attracted extensive interest [13]. Increasing evidence demonstrate that FTO promotes the growth and metastasis of several types of cancer, including gastric cancer [14,15], renal cell carcinoma [16], pancreatic cancer [17,18], esophageal cancer [19,20], colorectal cancer [21], multiple myeloma [22], head and neck squamous cell carcinoma [23], bladder cancer [24,25], endometrial cancer [26], liver cancer [27], lung cancer [28,29], breast cancer [30], and acute myeloid leukemia [31], among others.

Gynecologic cancers start in woman’s reproductive organs, including cervical cancer, ovarian cancer, uterine cancer, vaginal cancer, and vulvar cancer. So far, increasing evidence demonstrates that FTO plays an oncogenic role in gynecological cancer, including cervical and ovarian cancer. For example, FTO is overexpressed in human cervical cancer tissues and facilitates the proliferation, migration, and invasion of human cervical cancer cells [32,33]. In ovarian cancer, m^6^A RNA demethylases trigger the pathogenesis of ovarian cancer [34,35] and promote ovarian metastasis [36]. These studies emphasize the critical impact of abnormal FTO function on human diseases, especially in cancer. Therefore, targeted inhibition of FTO may provide a promising option for treating patients with gynecological cancer.

RNA demethylase inhibitors have been used in several experimental models and preclinical studies and demonstrate that FTO can work as a potential drug target against cancers [13,31,37,38]. Notably, treatments of tumor cells with RNA demethylase inhibitors have multiple cellular effects, including cell cycle arrest, apoptosis, differentiation and senescence, immune evasion, cancer stem cell self-renewal, epithelial–mesenchymal transition (EMT) pathway, and PI3K/AKT [31,37,39,40]. However, the role of RNA demethylases in the pathogenesis of uterine cancer, uLMS, is entirely unknown. Therefore, we hypothesized that RNA demethylases are dysregulated in uterine cancers and may play a crucial role in the pathogenesis of uLMS. In this study, we assessed the expression pattern of RNA demethylases in uLMS and myometrium tissues and characterized the role and mechanism of FTO in the pathogenesis of uLMS. Deep diving into the molecular mechanism of uLMS pathogenesis linking to RNA epigenetics would help improve these discriminated patients’ clinical management and health outcomes.

## 2. Results

### 2.1. The Expression Levels of FTO and ALKBH5 m^6^A RNA Demethylases Are Upregulated in uLMS Tissues Compared to Adjacent Myometrium from Women with uLMS

**Figure 1:**
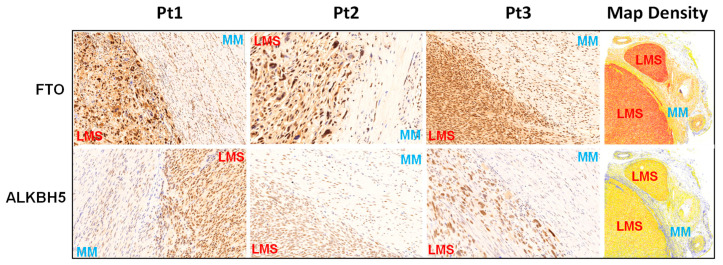
IHC staining of FTO and ALKBH5 in human uLMS tissues and adjacent myometrium. IHC staining for FTO and ALKBH5 is presented with three representative cases. The right column shows the density map of FTO and ALKBH5 for the same representative case. Blue color: negative; yellow color: low expression; brown color: moderate expression; red color: strong expression.

**Figure 2:**
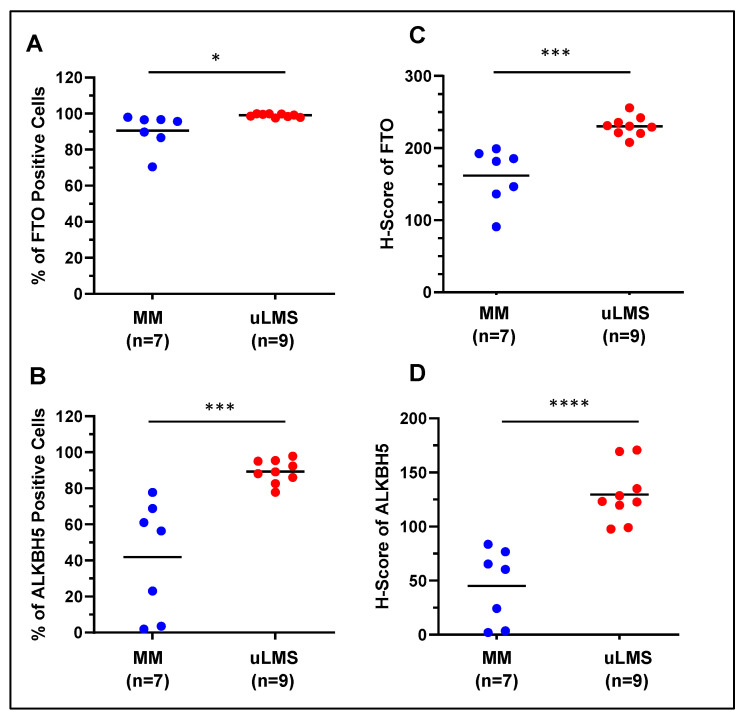
Percentage of FTO- and ALKBH5-positive cells and H-score of RNA demethylase levels in uLMS vs. myometrium: (**A**,**B**) percentage of FTO- and ALKBH5-positive cells in uLMS and myometrium tissues; (**C**,**D**) H-score of FTO and ALKBH5 in uLMS and myometrium tissues. * *p* < 0.05, *** *p* < 0.001, **** *p* < 0.0001. ns: no significant difference.

To determine the differential levels of RNA demethylase proteins between uLMS and MM (myometrium), IHC staining for FTO and ALKBH5 was performed. Figure 1 and Figure 2 show that the FTO- and ALKBH5-positive cells were significantly higher in uLMS than MM. The H-score of FTO and ALKBH5 was also significantly increased in uLMS (*n* = 9) compared to MM (*n* = 7). These studies indicated the critical role of RNA methylation erasers in the pathogenesis and progression of uLMS. Figure 2 (right column) revealed an increase in the expression density of FTO and ALKBH5 in uLMS compared to MM.

### 2.2. Inhibition of FTO Decreased the Cell Proliferation in uLMS Cells

**Figure 3:**
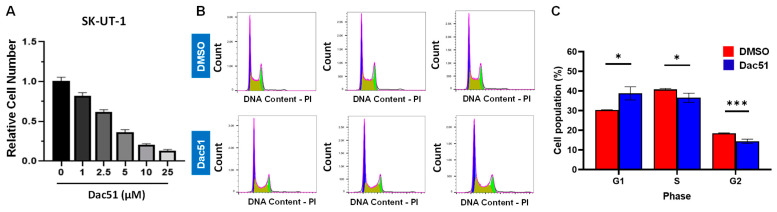
Treatment with Dac51 decreases uLMS cell proliferation and induces cell cycle arrest. (**A**) cell proliferation of SK-UT-1 cells in the presence or absence of Dac51; (**B**) flow cytometric analysis was performed to measure the cell cycle phase distribution in SK-UT-1 cells in the presence or absence of Dac51; (**C**) quantitative analysis of cell population in response to Dac51 treatment * *p* < 0.05; *** *p* < 0.001.

Dac51, as a small, potent FTO inhibitor, has been shown to inactivate the activity of FTO and synergize with the checkpoint blockade for better tumor control [41]. Therefore, we selected Dac51 for our in vitro cell model to assess its effect on uLMS cell growth. The trypan blue exclusion assay was performed in the SK-UT-1 cell line treated with dose ranges from 1 to 25 µM. Treatment with FTO inhibitor (Dac51) for 48 h showed a dose-dependent inhibitory effect on the proliferation of SK-UT-1 cells (Figure 3A).

### 2.3. Inhibition of FTO Induces Cell Cycle Arrest in uLMS Cells

Dac51 treatment resulted in the increased accumulation of cells in the G1 phase and a corresponding decrease in the S phase, indicating the blockade of G1 progression (Figure 3B,C). The percentage of cells in the G1 phase increased from 30.3% to 41.5% in response to 5 µM Dac51 treatment. Accordingly, the percentage of cells in the S phase decreased from 40.8% to 35.2% in response to the Dac51 treatment. These results suggest that Dac51 treatment suppressed uLMS proliferation via cell cycle arrest.

### 2.4. Dac51 Alters the Transcriptome of uLMS Cells

**Figure 4:**
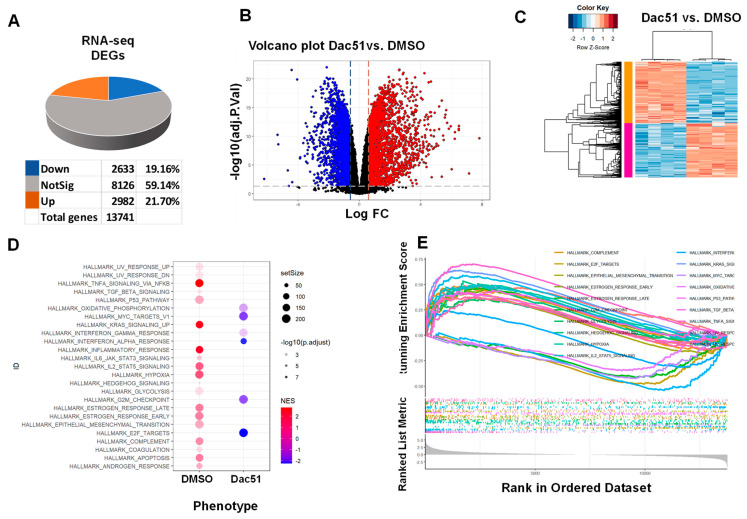
Treatment with Dac51 sculpts the transcriptome of uLMS cells. (**A**) Dac51-induced DEGs. DEGs were identified by voom + Limma at adjusted *p*-value cut off 0.05 and −1.5 > log2FC > 1.5; (**B**) volcano plots of the gene expression profiles of Dac51 vs. control; (**C**) heatmap; Pearson correlation was used to cluster DEG (Dac51 vs. control), which were then represented as a heatmap with the data scaled by Z score for each row; (**D**) hallmark analysis demonstrated the alteration of multiple pathways in SK-UT-1 cells in response to Dac51 treatment; (**E**) GSEA for comparison using the hallmark MSigDB collection. DEGs: differentially expressed genes, FC: fold change.

To characterize the Dac51-induced transcriptional changes in uLMS cells, RNA-sequencing analysis was performed in control (DMSO, *n* = 4) and Dac51-treated uLMS cells (*n* = 4). Dac51 treatment yielded 5615 DEGs (2633 down, 2982 up). Dac51 treatment upregulated 21.7% of gene expression and downregulated 19.16% of gene expression (Figure 4A). Differential gene expression analysis was performed by limma-voom. Figure 4B reveals the distribution of DEGs between the Dac51 treatment and DMSO control groups. Figure 4C exhibits distinct expression patterns between Dac51 treated vs. DMSO groups. Notably, the Dac51 treatment did not significantly alter the expression of RNA demethylase genes, including *FTO* and *ALKBH5*, since Dac51 exerted an inhibitory activity on FTO demethylase activity [41] rather than transcriptional activity on FTO.

#### 2.4.1. Enrichment Pathway Analysis

**Figure 5:**
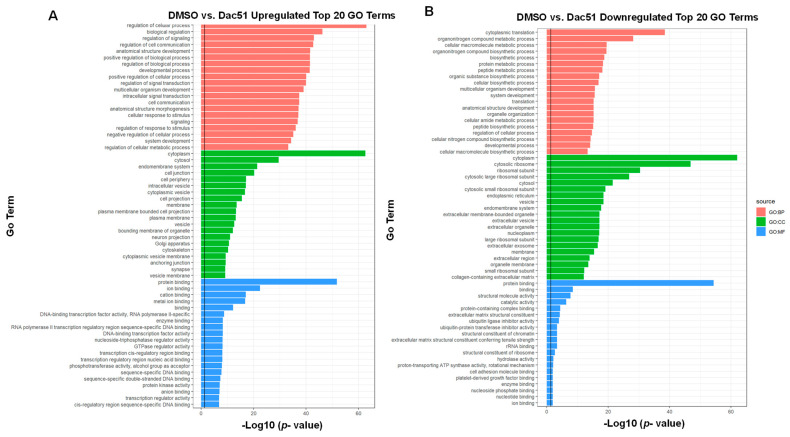
The GO functional annotation and pathway enrichment analysis of DEGs between DMSO and DAC-51 groups: (**A**) Upregulated gene enrichment in GO. (**B**) Downregulated gene enrichment in GO. GO, Gene Ontology; BP, biological process; CC, cellular component; MF, molecular function. DEGs were identified by voom at adjusted *p*-value cut off 0.05 and −1.5 > log2FC > 1.5.

Gene ontology analysis exhibited an enriched functional gene list of interest using g:Profiler (https://biit.cs.ut.ee/gprofiler/, accessed on 2 December 2022). We demonstrated that several gene sets were enriched in Dac51 vs. control group, including xenobiotic metabolism, UV response, TNFa signaling via NFkB, P53 pathway, MYC targets, MTORC1 spindle, KRAS signaling, inflammatory response, G_2_M checkpoint, and E2F targets, among others (Figure 4D,E). To gain further insight into the changes by FTO inhibition, the biological classification analysis of DEGs was performed using the DAVID database (https://david.ncifcrf.gov/, accessed on 2 December 2022), including functional and pathway enrichment analyses. Sorting by *p*-value, the top twenty GO terms of the BP, MF, and CC categories are shown in Figure 5. The upregulated genes were mainly involved in regulating the cellular process, biological process, and signaling in the BP category, mainly constituting the cytoplasm in the CC category and protein binding in the MF category. The downregulated genes were mainly associated with cytoplasmic translation and the organonitrogen compound and cellular macromolecular metabolic process in the BP category, constituted the cytoplasm in the CC category, and were associated with protein binding and catalytic activity in the MF category. Figure S1 shows the results of the Reactome pathway analysis of the RNA-seq data. The top five pathways with up-DEGs genes in response to Dac51 treatment in the SK-UT-1 cells were signaling transduction, signaling by receptor tyrosine kinase, effects of PIP2 hydrolysis, death receptor signaling, and interleukin-10 signaling. In contrast, the top five pathways with the downregulation of genes in response to Dac51 treatment included eukaryotic translation elongation, peptide chain elongation, formation of a pool of free 40 S subunits, nonsense-mediated decay independent of exon junction complex, and eukaryotic translation termi nation.

#### 2.4.2. The Expression of Cell Cycle-Related Genes Is Altered upon Dac51 Treatment

**Figure 6:**
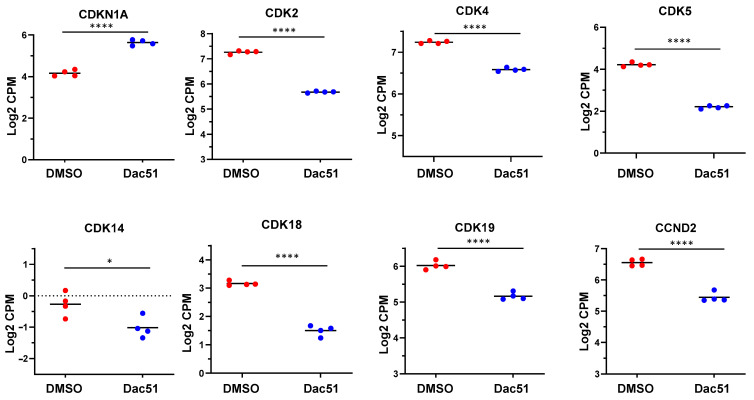
Dac51 altered cell cycle-related gene expression in uLMS cells. RNA-seq revealed the upregulation of *CDKN1A* and downregulation of *CDK2*, *CDK4*, *CDK5*, *CDK14*, *CDK17*, *CDK18*, and *CDK19* in uLMS cells. * *p* < 0.05; **** *p* < 0.0001.

To determine the molecular mechanism underlying Dac51-induced suppression of cell cycle progression in uLMS cells, we compared the expression levels of cell-cycle-related genes between control and Dac51-treated cells. As shown in Figure 6, Dac51 treatment increased the expression of *CDKN1A* and reduced the expression of *CDK2*, *CDK4*, *CDK5*, *CDK14*, *CDK18*, *CDK19*, and *CCND2*. Since *CDKN1A* and *CDK* members were critical in cell cycle progression [42,43,44], our results suggested that these cell progression regulators may play an essential role in DAC51-induced cell cycle arrest. In addition, we validated the expression of two key cell-cycle-related genes (*CDKN1A* and *CDK2*) by q-PCR. The data were consistent with RNA-seq data (Figure S2).

#### 2.4.3. Dac51 Altered the Gene Expression Associated with Transcriptional Factors

Transcriptional factors play an important role in many biological processes, and their control is disrupted in cancer cells [45]. It was reported that *FTO* knockdown and inhibition repressed the E2F targets [37]. The dysregulation of these core TFs interconnected transcriptional loops to establish and reinforce the abnormal gene expression program in cancer cells [46]. To determine the transcriptional factors involved in the cell cycle progression in response to Dac51 treatment, we employed Ingenuity Pathway Analysis to determine the potential regulatory mechanism of E2F and SP1 transcription regulators that may regulate cell-cycle-related genes. We filtered our dataset for upstream regulators that target *CDK2*, *CDK4*, *CDK5*, and *CCND2*. We demonstrated that E2F1 is the upstream regulator for *CDK2*, *CDK4*, *CDK5*, and *CCND2*. In addition, E2F2 is the upstream regulator for *CDK2*, E2F4 is the upstream regulator targeting *CDK2* and *CDK4*. Notably, SP1 is the common upstream regulator that targets *CDK2*, *CDK4*, and *CCND2*. These analyses demonstrate that Dac51 treatment may alter the expression of cell-cycle-related genes via transcription regulators, such as E2F and SP1, which may contribute to the inhibitory effect of the FTO inhibitor on cell cycle and proliferation. 

To understand the thematic association between TFs and transcriptional changes in response to the Dac51 treatment, we performed gene list enrichment analysis using Enrichr and TF enrichment analysis using the Encode/ChEA database. As a result, we identified *NR3C1*, *SUZ12*, *ZNF*, *MBD3*, and *LEF1* among the most enriched TFs with up-DEGs (Table S1). In addition, ChEA with down-DEGs identified *MYC*, *EGR1*, *E2F1*, *XRN2*, and *CCND1* among the most enriched TFs (Table S2). 

#### 2.4.4. Dac51 Treatment Altered the Expression of Epigenetic Regulators

**Figure 7:**
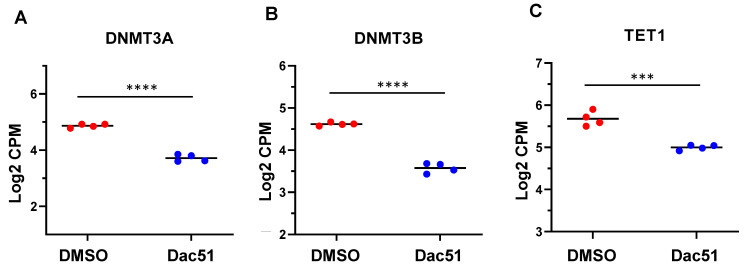
Dac51 altered DNA methylation-related genes in uLMS cells. RNA-seq revealed the downregulation of *DNMT3A* (**A**), *DNMT3B* (**B**), and *TET1* (**C**) in uLMS cells in response to Dac51 treatment. *** *p* < 0.001; **** *p* < 0.0001.

It has been reported that the dynamic interplay between DNA and RNA modification plays a crucial role in orchestrating numerous biological processes [47,48,49]. Therefore, we performed targeted gene analysis using our RNA-seq data and demonstrated that the expression of genes that regulated the dynamic status of DNA methylation was modulated in Dac51-treated SK-UT-1 cells. These DNA methylation/demethylation-related DEGs included *DNMT3A*, *DNMT3B*, and *TET1* (Figure 7A–C).

**Figure 8:**
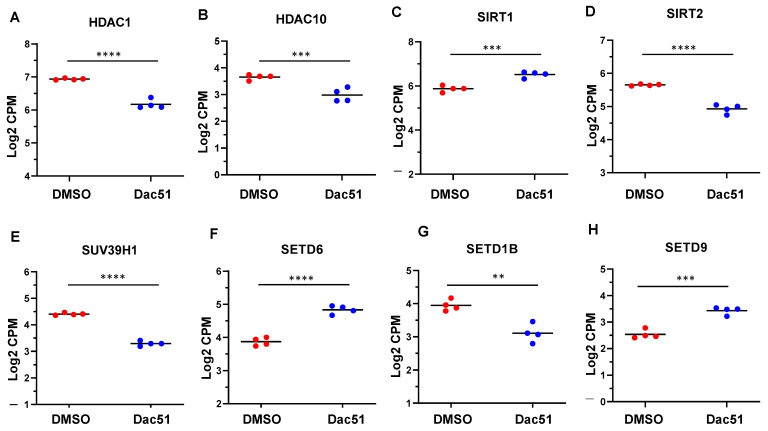
Dac51 altered the expression levels of histone modification-regulated genes in uLMS cells. RNA-seq revealed the altered expression of *HDAC1* (**A**), *HDAC10* (**B**), *SIRT1* (**C**), *SIRT2* (**D**), *SUV39H1* (**E**), *SETD6* (**F**), *SETD1B* (**G**), and *SETD9* (**H**) in uLMS cells in response to Dac51 treatment. ** *p* < 0.01; *** *p* < 0.001; **** *p* < 0.0001.

To determine the relationship between RNA methylation and histone acetylation, we characterized the genes related to histone acetylation in SK-UT-1 cells after treatment with Dac51. As shown in Figure 8A–D, the inhibition of FTO by Dac51 significantly modulated the expression of *HDAC1*, *HDAC10*, *SIRT1*, and *SIRT2*.

We also revealed that Dac51 treatment modulated the expression levels of several genes encoding histone methylation enzymes. These genes included *SUV39H1*, *SETD6*, *SETD1B*, and *SETD9* (Figure 8E–H). We also validated the expression of several epigenetic genes, including *SIRT1*, *SIRT2*, *HDAC10*, and *SUV39H1*, by q-PCR. The data were consistent with RNA-seq analysis (Figure S2). These analyses suggest that Dac51 treatment may alter the transcriptome via epigenetic mechanisms.

#### 2.4.5. Dac51 Altered the Gene Expression Correlating with microRNA Regulation

We used TargetScan microRNA analysis in the Enrichr web server (https://maayanlab.cloud/Enrichr/, accessed on 15 December 2022) to determine the association of miRNAs with the input query gene set. The top 20 enriched human miRNAs are displayed based on the −log10 (*p*-value). As shown in Tables S3 and S4, the miRNAs at the top have the most significant overlap with the up-DEGs or down-DEGs, respectively. 

## 3. Discussion

Uterine LMS is a highly aggressive tumor with high rates of tumor recurrence, progression, and metastasis [4]. The origin and molecular mechanism underlying and driving its clinical and biological behavior remain unclear [5]. Although the dysregulation of FTO contributing to tumorigenesis via an m^6^A-dependent mechanism has been identified, the role of FTO in uLMS is entirely unknown. In this study, we demonstrated for the first time that RNA demethylases FTO and ALKBH5 were aberrantly upregulated in uLMS and exhibited an essential tumor-promoting role in uLMS. FTO could reprogram the oncogenic and specific FTO inhibitors, such as Dac51, exhibit promising therapeutic efficacy in treating uLMS. 

Abnormal cell proliferation via decreasing cell cycle arrest and apoptosis is common in many cancers [50,51,52,53]. We previously revealed that uLMS cells grow faster than myometrial cells [54]. In this study, we revealed that the inhibition of FTO with Dac51 decreased uLMS proliferation in a dose-dependent manner via cell cycle arrest. Notably, FTO as a therapeutic target has been reported in many types of cancers and experimental models. Accordingly, several FTO inhibitors have been developed, including GS1 and GS2 [31], 18077 and 18097 [13], 13a [55], C6 [39], Rhein [56], FB23-2 [16], and Dac51 [41]. The latter showed a potent inhibitory effect as an FTO inhibitor. Furthermore, these pharmacological inhibitors showed an anti-tumor effect with decreased cancer phenotype upon suppressing FTO m^6^A demethylase activity [28,29]. All of these studies demonstrated the critical role of RNA demethylases in cancer development, and the targeted inhibition of FTO showed beneficial effects in many types of neoplasms, including gynecological cancer. Our studies revealed the oncogenic role of RNA demethylases in uLMS, which were previously identified in other gynecological cancers, including cervical and ovarian cancer. 

To further determine the mechanisms associated with Dac51-induced inhibition, we performed a genome-wide RNA-sequencing experiment comparing the profiles of DMSO-treated with Dac51-treated uLMS cells. The transcriptome analysis revealed that the targeted inhibition of FTO with Dac51 altered several critical biological pathways that may contribute to uLMS pathogenesis. Notably, we demonstrated that the inhibitory effect of Dac51 on uLMS cells is consistent with the altered expression levels of cell cycle-related genes, concomitantly with the increased expression of *CDKN1A* and the decreased expression of *CDK2*, *CDK4*, *CDK5*, *CDK14*, *CDK18*, *CDK19*, and *CCND2*. Therefore, targeting FTO with the small inhibitor Dac51 suppressed uLMS proliferation and induced cell cycle arrest via CDK members and other cell-cycle-related proteins.

TFs play a central role in cancer progression. Some TFs alter multiple biological processes, including DNA repair genes, cell proliferation, clonal heterogeneity of the disease, cellular stresses, and therapy resistance. TFs with co-activators and co-repressors caused alterations in gene expression at specific sites in the genome [57]. By ChEA analysis, we demonstrated the altered network of TFs based on the overlapping targets and binding site proximity, which may partially explain why the Dac51 treatment contributed to the suppression of uLMS. For example, MYC is a key TF that plays an essential role in cancer cell proliferation and survival and has an impact on tumor progression and therapy resistance [58,59,60]. Our study showed that targeting FTO exhibited an association between down-DEGs and MYC targets, providing a mechanism to alter the MYC-driven gene expression without directly targeted MYC. E2F1 in our top list is a well-recognized regulator of the cell cycle and a potent mediator of DNA-damage-induced apoptosis and checkpoint response [61] and has been reported to play an important role in promoting various cancers [62,63,64]. In addition, E2F1 is reported to be involved in uterine cancer progression [65,66,67]. Notably, E2F1, its binding partner TFDP1, and E2F1 downstream effectors in LMS were significantly upregulated compared to normal muscle [68]. Our Ingenuity Pathway Analysis demonstrated that E2F1 is the upstream regulator targeting CDK2, CDK4, CDK5, and CCND2. Therefore, Dac51-induced enrichment of a positive association between E2F1 and its downstream genes suggests the important role that FTO plays in controlling the transcriptional program via the E2F-regulated pathway in LMS. Our studies are consistent with previous findings that the targeted inhibition of FTO with FB23 and FB23-2, or FTO knockdown, repressed the MYC and E2F targets [37]. SP1, as a transcriptional factor, plays an important role in cancer development [69] and regulates cell cycle progression [70,71,72]. In this study, SP1 is the common upstream regulator that targets CDK2, CDK4, and CCND2. Therefore, one may consider that FTO may disrupt the normal cell cycle process in uLMS cells by altering the TF-driven pathways.

Notably, the interplay between RNA methylation and chromatin regulation has been reported [73,74,75]. N (6)-methyladenosine of chromosome-associated regulatory RNA regulates chromatin state and transcription [76]. The recent study by Wei J et al. demonstrated that FTO mediates LINES m^6^A demethylation and chromatin regulation in mESCs and mouse development. In this study, our results revealed that targeted inhibition of FTO altered the expression of histone acetylation modulators, histone methylation enzymes, and DNA-methylation-related epigenetic regulators, further demonstrating the strong interconnection between RNA epigenetics and other epigenetic mechanisms. Our studies indicated that the targeted inhibition of FTO might alter the transcriptome via reprogramming the network of oncogenic epigenomes in uLMS. In the future, it is necessary to understand other m^6^A regulators, including m^6^A writers and readers, that may contribute to the occurrence and progression of uLMS through the dynamic RNA methylation mechanism. 

**Figure 9:**
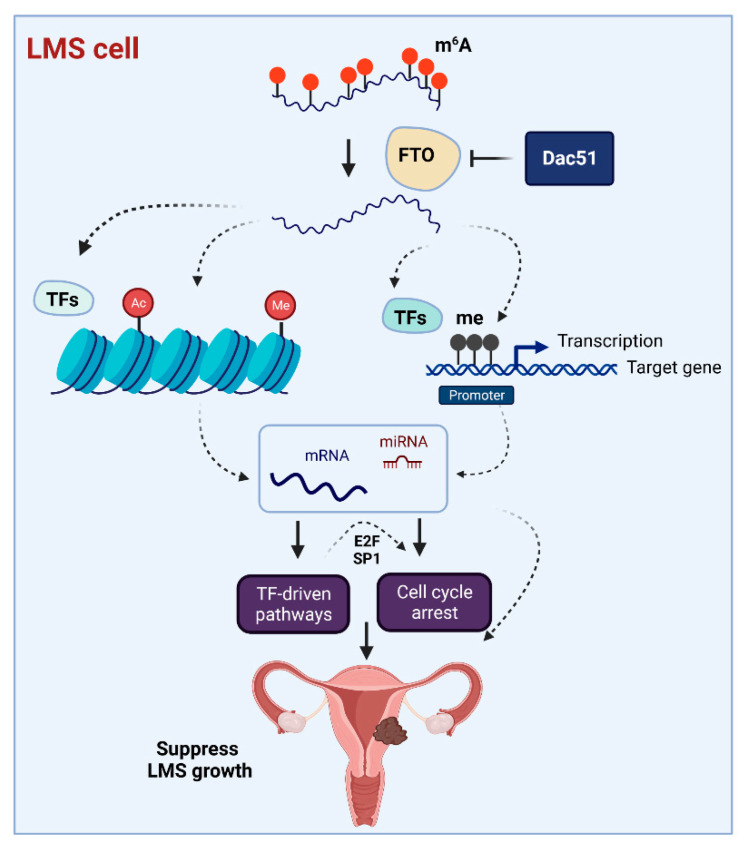
Experimental model. The model shows that targeting FTO with Dac51 induces cell cycle arrest and alters the TF network as well as interactions between target genes and epigenetic/miRNA regulators in uLMS cells. In addition, TFs, such as E2F members and SP1, are the upstream regulators targeting CDK members, which modulate cell cycle progression. This figure was created using BioRender software online app (BioRender.com).

As per our studies, we proposed a mechanism model for the targeted inhibition of FTO in uLMS based on our novel findings that (1) RNA demethylases FTO and ALKBH5 are dysregulated in uLMS tumors; (2) targeting FTO with Dac51 alters the uLMS phenotype with a decrease in cell proliferation and a modulation of cell-cycle-related genes and others; (3) Dac51 reversed the phenotype of uLMS via different biological pathways including TF-driven signaling; (4) RNA demethylases constitute a distinguished vulnerability in malignant uLMS, and FTO inhibitors, such as Dac51, alter key pathways and reprogram the oncogenic profiling and miRNA network to suppress the uLMS phenotype (Figure 9).

In conclusion, our study demonstrated for the first time that uLMS tumors exhibited an aberrant upregulation of m^6^A RNA demethylase proteins, highlighting the important role of m^6^A RNA methylation in the pathogenesis of uLMS. Targeted inhibition of RNA m^6^A demethylase FTO may impart beneficial effects in uLMS and provide a promising and novel strategy for treating patients with this aggressive uterine cancer. 

## 4. Materials and Methods

### 4.1. Uterine Leiomyosarcoma Samples

The uLMS tissues were obtained from the University of Chicago Tissue Bank. Approval from the Institutional Review Board (# 20-1414) at the University of Chicago was obtained for the retrospective chart review of uLMS patients. Informed consent was obtained from all the study participants before surgery. The cases with an initial diagnosis of uLMS at the University of Chicago Hospital were reviewed, and the diagnosis was confirmed by hematoxylin–eosin (H&E) evaluation and immunohistochemistry. A total of nine cases with uterine uLMS were used as previously described [77].

### 4.2. Immunohistochemistry

Immunohistochemistry (IHC) was performed as described previously [77]. The primary antibodies FTO (Abcam, Ab109411, Cambridge, UK) and ALKBH5 (Abcam, Ab 32117) were used for IHC. To determine the percentage and intensity of FTO- and ALKBH5-positive cells, QuPath software (version 0.2.3) (https://qupath.github.io, accessed on 15 November 2022) was used with the positive cell detection command. Thresholds were set to categorize cells according to nuclei staining intensity: negative, weak, moderate, and strong intensity. The histochemical scoring (H-score) captures both the intensity and the proportion of the FTO/ALKBH5-positive cells from the IHC image and comprises values between 0 and 300 [78], thereby offering a dynamic range to quantify FTO/ALKBH5 abundance between myometrium and uLMS. Human testis tissues were used as positive tissues for FTO and ALKBH5 staining.

### 4.3. Cells and Reagents

The culture condition of human uterine leiomyosarcoma cell line SK-UT-1 was described previously [77].

FTO inhibitor Dac51 was purchased from Selleck Chemical (Cat# S9876, Houston, TX, USA). The range of doses tested was 1–25 µM.

### 4.4. Proliferation Assay

A trypan blue exclusion assay was performed for cell proliferation measurement. Cells were seeded into 12-well tissue culture plates and treated with the Dac51 at a dose range of 1–25 µM for 48 h. An equal amount of DMSO was used as vehicle control. After treatment, the cells were trypsinized and collected by centrifuge. The cells were resuspended in a serum-free medium. An equal volume of 0.4% trypan blue and cell suspension was mixed and applied to a hemacytometer for cell counting. Viable cells were unstained. This assay was performed in triplicate.

### 4.5. Measurement of Cell Cycle Phase Distribution

Cell cycle distribution was determined by flow cytometric analysis. Briefly, SK-UT-1 cells were cultured in the medium containing 5 µM of Dac51 for 48 h. Control cells were cultured in a medium containing equal amount of DMSO. Cells were then washed with PBS, fixed in 70% ethanol for at least 30 min, and hypotonically lysed in 0.2 mL of DNA staining solution (0.05 mg/mL PI (Sigma, St. Louis, MO, USA) and 0.1% Triton X-100). The cell cycle data were analyzed with an Epics XL-MCL flow cytometer (Beckman Coulter, Miami, FL, USA), with System II (version 3.0) software (Beckman Coulter). Additional analysis of cell cycle distribution was determined by using Modfit LT (Topsham, ME, USA).

### 4.6. RNA-Sequencing

The uLMS cell line (SK-UT-1) was treated with 5 µM Dac51 for 48 h. Cells were subjected to RNA isolation using Trizol. RNA and library quality and quantity were assessed as described previously [77]. An Illumina NovaSEQ6000 was used for library sequencing. 

### 4.7. Transcriptome Profiles Analysis

#### 4.7.1. Transcriptome Data Analysis

The classical alignment-based mapper STAR, version 2.6.1d (GitHub, Inc., San Francisco, CA, USA) (23) was used to map sequencing reads to a human reference transcriptome. The results of STAR mapping were quantified by Salmon, version 1.4.0. Then, Bioconductor (https://bioconductor.org/packages/release/bioc/html/tximport.html, accessed on 2 December 2022) was used to read Salmon outputs into the R environment. Downstream analyses were performed as described previously [77].

#### 4.7.2. Differential Gene Expression Analysis

To identify the differentially expressed genes (DEGs) between treatment and control groups, the algorithm was implemented in R packages Limma + voom [79]. We used a cutoff of −1.5 > fold change > 1.5 and a *p*-value of 0.05. In addition, Benjamini and Hochberg’s (BH) method was performed to control the false discovery rate of all the genes with adjusted *p*-values less than 0.05.

#### 4.7.3. Gene List Enrichment Analysis

Comprehensive gene set enrichment analysis for regulation machinery was carried out using the Enrichr (version 3.1) [80] package in R (https://maayanlab.cloud/Enrichr/ (accessed on 2 December 2022). We used ChEA and TargetScan microRNA in Enrichr to determine the mechanisms underlying the regulation of DEGs. We also used Ingenuity Pathway Analysis (IPA) (Qiagen) to determine the link between TFs and cell-cycle-related genes. The cutoffs of IPA are −1 to +1 for logFC, and 0.01 for adj. *p*. val, respectively.

### 4.8. cDNA Synthesis and Quantitative Real-Time Polymerase Chain Reaction

Total cellular RNA was isolated from frozen pellets using the PureLink RNA Mini Kit (Ambion, Waltham, MA, USA). RNA integrity was measured by an Agilent bioanalyzer. cDNA synthesis was performed in 20 µL reaction volume using RNA to cDNA EcoDry Premix (Takara Bio USA, San Jose, CA, USA). The reaction mixture was incubated for 1 h at 42 °C and stopped by incubation at 70 °C for 10 min.

Quantitative real-time polymerase chain reaction (qRT-PCR) was performed to measure the relative mRNA expression of genes listed in Table S5. Primers were purchased from Integrated DNA Technologies (Coralville, IA, USA). An equal amount of cDNA from each sample was added to the Mastermix containing appropriate primer sets and SYBR green supermix (Bio-Rad, Hercules, CA, USA) in a 20 µL reaction volume. All samples were analyzed in triplicate. Real-time PCR analyses were performed using a Bio-Rad CFX96. Cycling conditions include denaturation at 95 °C for 2 min followed by 40 cycles of 95 °C for 10 s and 60 °C for 30 s, followed by 65 °C for 5 s. Synthesis of a DNA product of the expected size was confirmed by melting curve analysis. The expression of 18 S ribosomal RNA was used as an endogenous control to normalize the expression data. Negative control was performed by running the reaction without cDNA template. The relative RNA expression was expressed as fold changes and calculated using the 2^∆∆CT^ method.

### 4.9. Statistical Analysis

A comparison of the two and multiple groups was carried out as described previously [77]. Data were presented as mean ± standard error (SE), and the significant difference was defined as *p* < 0.05.

## Data Availability

Raw FASTQ files have been requested to be deposited in the NCBI Gene Expression Omnibus (GSE 224306).

## Supplementary Materials

The following supporting information can be downloaded at https://www.mdpi.com/article/10.3390/ijms24097957/s1.

## References

[1] D’Angelo E., Prat J. (2010). Uterine sarcomas: A review. Gynecol. Oncol..

[2] Seagle B.-L.L., Sobecki-Rausch J., Strohl A.E., Shilpi A., Grace A., Shahabi S. (2017). Prognosis and treatment of uterine leiomyosarcoma: A National Cancer Database study. Gynecol. Oncol..

[3] Hensley M.L., Blessing J.A., Mannel R., Rose P.G. (2008). Fixed-dose rate gemcitabine plus docetaxel as first-line therapy for metastatic uterine leiomyosarcoma: A Gynecologic Oncology Group phase II trial. Gynecol. Oncol..

[4] Gadducci A., Landoni F., Sartori E., Zola P., Maggino T., Lissoni A., Bazzurini L., Arisio R., Romagnolo C., Cristofani R. (1996). Uterine Leiomyosarcoma: Analysis of Treatment Failures and Survival. Gynecol. Oncol..

[5] De Almeida B.C., dos Anjos L.G., Dobroff A.S., Baracat E.C., Yang Q., Al-Hendy A., Carvalho K.C. (2022). Epigenetic Features in Uterine Leiomyosarcoma and Endometrial Stromal Sarcomas: An Overview of the Literature. Biomedicines.

[6] Yang Q., Ciebiera M., Bariani M.V., Ali M., Elkafas H., Boyer T.G., Al-Hendy A. (2021). Comprehensive Review of Uterine Fibroids: Developmental Origin, Pathogenesis, and Treatment. Endocr. Rev..

[7] Chen X.-Y., Zhang J., Zhu J.-S. (2019). The role of m6A RNA methylation in human cancer. Mol. Cancer.

[8] Qiu W., Zhou Y., Wu H., Lv X., Yang L., Ren Z., Tian H., Yu Q., Li J., Lin W. (2021). RNA Demethylase FTO Mediated RNA m6A Modification Is Involved in Maintaining Maternal-Fetal Interface in Spontaneous Abortion. Front. Cell Dev. Biol..

[9] Schwartz S., Mumbach M.R., Jovanovic M., Wang T., Maciag K., Kushkin G.G., Mertins P., Ter-Ovanesyan D., Habib N., Cacchiarelli D. (2014). Perturbation of m6A Writers Reveals Two Distinct Classes of mRNA Methylation at Internal and 5′ Sites. Cell Rep..

[10] Knuckles P., Carl S.H., Musheev M., Niehrs C., Wenger A., Bühler M. (2017). RNA fate determination through cotranscriptional adenosine methylation and microprocessor binding. Nat. Struct. Mol. Biol..

[11] Shi H., Wang X., Lu Z., Zhao B.S., Ma H., Hsu P.J., Liu C., He C. (2017). YTHDF3 facilitates translation and decay of *N*^6^-methyladenosine-modified RNA. Cell Res..

[12] Wang X., Zhao B.S., Roundtree I.A., Lu Z., Han D., Ma H., Weng X., Chen K., Shi H., He C. (2015). N6-methyladenosine Modulates Messenger RNA Translation Efficiency. Cell.

[13] Xie G., Wu X.-N., Ling Y., Rui Y., Wu D., Zhou J., Li J., Lin S., Peng Q., Li Z. (2021). A novel inhibitor of N6-methyladenosine demethylase FTO induces mRNA methylation and shows anti-cancer activities. Acta Pharm. Sin. B.

[14] Zhou Y., Wang Q., Deng H., Xu B., Zhou Y., Liu J., Liu Y., Shi Y., Zhang X., Jiang J. (2022). N6-methyladenosine demethylase FTO promotes growth and metastasis of gastric cancer via m(6)A modification of caveolin-1 and metabolic regulation of mitochondrial dynamics. Cell Death Dis..

[15] Wang D., Qu X., Lu W., Wang Y., Jin Y., Hou K., Yang B., Li C., Qi J., Xiao J. (2021). N6-Methyladenosine RNA Demethylase FTO Promotes Gastric Cancer Metastasis by Down-Regulating the m6A Methylation of ITGB1. Front. Oncol..

[16] Xu Y., Zhou J., Li L., Yang W., Zhang Z., Zhang K., Ma K., Xie H., Zhang Z., Cai L. (2022). FTO-mediated autophagy promotes progression of clear cell renal cell carcinoma via regulating SIK2 mRNA stability. Int. J. Biol. Sci..

[17] Wang W., He Y., Wu L., Zhai L.L., Chen L.J., Yao L.C., Yu K.H., Tang Z.G. (2022). N(6)-methyladenosine RNA demethylase FTO regulates extracellular matrix-related genes and promotes pancreatic cancer cell migration and invasion. Cancer Med..

[18] Tan Z., Shi S., Xu J., Liu X., Lei Y., Zhang B., Hua J., Meng Q., Wang W., Yu X. (2022). RNA N6-methyladenosine demethylase FTO promotes pancreatic cancer progression by inducing the autocrine activity of PDGFC in an m6A-YTHDF2-dependent manner. Oncogene.

[19] Duan X., Yang L., Wang L., Liu Q., Zhang K., Liu S., Liu C., Gao Q., Li L., Qin G. (2022). m6A demethylase FTO promotes tumor progression via regulation of lipid metabolism in esophageal cancer. Cell Biosci..

[20] Liu S., Huang M., Chen Z., Chen J., Chao Q., Yin X., Quan M. (2020). FTO promotes cell proliferation and migration in esophageal squamous cell carcinoma through up-regulation of MMP13. Exp. Cell Res..

[21] Wang J., Qiao Y., Sun M., Sun H., Xie F., Chang H., Wang Y., Song J., Lai S., Yang C. (2022). FTO promotes colorectal cancer progression and chemotherapy resistance via demethylating G6PD/PARP1. Clin. Transl. Med..

[22] Xu A., Zhang J., Zuo L., Yan H., Chen L., Zhao F., Fan F., Xu J., Zhang B., Zhang Y. (2021). FTO promotes multiple myeloma progression by posttranscriptional activation of HSF1 in an m6A-YTHDF2-dependent manner. Mol. Ther..

[23] Zhang Y., Chen L., Wu X., Sun Z., Wang F., Wang B., Dong P. (2021). The RNA N6-Methyladenosine Demethylase FTO Promotes Head and Neck Squamous Cell Carcinoma Proliferation and Migration by Increasing CTNNB1. Int. J. Gen. Med..

[24] Zhou G., Yan K., Liu J., Gao L., Jiang X., Fan Y. (2021). FTO promotes tumour proliferation in bladder cancer via the FTO/miR-576/CDK6 axis in an m6A-dependent manner. Cell Death Discov..

[25] Tao L., Mu X., Chen H., Jin D., Zhang R., Zhao Y., Fan J., Cao M., Zhou Z. (2021). FTO modifies the m6A level of MALAT and promotes bladder cancer progression. Clin. Transl. Med..

[26] Zhang L., Wan Y., Zhang Z., Jiang Y., Lang J., Cheng W., Zhu L. (2021). FTO demethylates m6A modifications in HOXB13 mRNA and promotes endometrial cancer metastasis by activating the WNT signalling pathway. RNA Biol..

[27] Ye Z., Wang S., Chen W., Zhang X., Chen J., Jiang J., Wang M., Zhang L., Xuan Z. (2020). Fat mass and obesity-associated protein promotes the tumorigenesis and development of liver cancer. Oncol. Lett..

[28] Li J., Han Y., Zhang H., Qian Z., Jia W., Gao Y., Zheng H., Li B. (2019). The m6A demethylase FTO promotes the growth of lung cancer cells by regulating the m6A level of USP7 mRNA. Biochem. Biophys. Res. Commun..

[29] Liu J., Ren D., Du Z., Wang H., Zhang H., Jin Y. (2018). m^6^A demethylase FTO facilitates tumor progression in lung squamous cell carcinoma by regulating MZF1 expression. Biochem. Biophys. Res. Commun..

[30] Niu Y., Lin Z., Wan A., Chen H., Liang H., Sun L., Wang Y., Li X., Xiong X.-F., Wei B. (2019). RNA N6-methyladenosine demethylase FTO promotes breast tumor progression through inhibiting BNIP3. Mol. Cancer.

[31] Su R., Dong L., Li Y., Gao M., Han L., Wunderlich M., Deng X., Li H., Huang Y., Gao L. (2020). Targeting FTO Suppresses Cancer Stem Cell Maintenance and Immune Evasion. Cancer Cell.

[32] Zou D., Dong L., Li C., Yin Z., Rao S., Zhou Q. (2019). The m6A eraser FTO facilitates proliferation and migration of human cervical cancer cells. Cancer Cell Int..

[33] Huang J., Yang J., Zhang Y., Lu D., Dai Y. (2023). FTO promotes cervical cancer cell proliferation, colony formation, migration and invasion via the regulation of the BMP4/Hippo/YAP1/TAZ pathway. Exp. Cell Res..

[34] Jiang Y., Wan Y., Gong M., Zhou S., Qiu J., Cheng W. (2020). RNA demethylase ALKBH5 promotes ovarian carcinogenesis in a simulated tumour microenvironment through stimulating NF-kappaB pathway. J. Cell Mol. Med..

[35] Zhao L., Kong X., Zhong W., Wang Y., Li P. (2020). FTO accelerates ovarian cancer cell growth by promoting proliferation, inhibiting apoptosis, and activating autophagy. Pathol. Res. Pract..

[36] Sun R., Yuan L., Jiang Y., Wan Y., Ma X., Yang J., Sun G., Zhou S., Wang H., Qiu J. (2023). ALKBH5 activates FAK signaling through m6A demethylation in *ITGB1* mRNA and enhances tumor-associated lymphangiogenesis and lymph node metastasis in ovarian cancer. Theranostics.

[37] Huang Y., Su R., Sheng Y., Dong L., Dong Z., Xu H., Ni T., Zhang Z.S., Zhang T., Li C. (2019). Small-Molecule Targeting of Oncogenic FTO Demethylase in Acute Myeloid Leukemia. Cancer Cell.

[38] Huff S., Kummetha I.R., Zhang L., Wang L., Bray W., Yin J., Kelly V., Wang Y., Rana T.M. (2022). Rational Design and Optimization of m^6^A-RNA Demethylase FTO Inhibitors as Anticancer Agents. J. Med. Chem..

[39] Qin B., Bai Q., Yan D., Yin F., Zhu Z., Xia C., Yang Y., Zhao Y. (2022). Discovery of novel mRNA demethylase FTO inhibitors against esophageal cancer. J. Enzym. Inhib. Med. Chem..

[40] You Y., Fu Y., Huang M., Shen D., Zhao B., Liu H., Zheng Y., Huang L. (2022). Recent Advances of m6A Demethylases Inhibitors and Their Biological Functions in Human Diseases. Int. J. Mol. Sci..

[41] Liu Y., Liang G., Xu H., Dong W., Dong Z., Qiu Z., Zhang Z., Li F., Huang Y., Li Y. (2021). Tumors exploit FTO-mediated regulation of glycolytic metabolism to evade immune surveillance. Cell Metab..

[42] Lee J., Kim K., Ryu T., Jung C.R., Lee M.S., Lim J.H., Park K., Kim D.S., Son M.Y., Hamamoto R. (2021). EHMT1 knockdown induces apoptosis and cell cycle arrest in lung cancer cells by increasing CDKN1A expression. Mol. Oncol..

[43] Li B., Li A., You Z., Xu J., Zhu S. (2020). Epigenetic silencing of CDKN1A and CDKN2B by SNHG1 promotes the cell cycle, migration and epithelial-mesenchymal transition progression of hepatocellular carcinoma. Cell Death Dis..

[44] He C., Chen H., Liu Y., Li X., Zhang C., Qin Q., Pang Q. (2020). miR-106b-5p promotes cell proliferation and cell cycle progression by directly targeting CDKN1A in osteosarcoma. Exp. Ther. Med..

[45] Bradner J.E., Hnisz D., Young R.A. (2017). Transcriptional Addiction in Cancer. Cell.

[46] Chen Y., Xu L., Lin R.Y.-T., Müschen M., Koeffler H.P. (2020). Core transcriptional regulatory circuitries in cancer. Oncogene.

[47] Lasman L., Hanna J.H., Novershtern N. (2020). Role of m6A in Embryonic Stem Cell Differentiation and in Gametogenesis. Epigenomes.

[48] Fiorenzano A., Pascale E., Gagliardi M., Terreri S., Papa M., Andolfi G., Galasso M., Tagliazucchi G.M., Taccioli C., Patriarca E.J. (2018). An Ultraconserved Element Containing lncRNA Preserves Transcriptional Dynamics and Maintains ESC Self-Renewal. Stem Cell Rep..

[49] Fiorenzano A., Pascale E., Patriarca E.J., Minchiotti G., Fico A. (2019). LncRNAs and PRC2: Coupled Partners in Embryonic Stem Cells. Epigenomes.

[50] Han F., Cheng C., Xu Q., Chen J., Yang Z., Liu J. (2022). DEPDC1B promotes colorectal cancer via facilitating cell proliferation and migration while inhibiting apoptosis. Cell Cycle.

[51] Chen Z., Tang W.J., Zhou Y.H., Chen Z.M., Liu K. (2021). Andrographolide inhibits non-small cell lung cancer cell proliferation through the activation of the mitochondrial apoptosis pathway and by reprogramming host glucose metabolism. Ann. Transl. Med..

[52] Luo D., Fan H., Ma X., Yang C., He Y., Ge Y., Jiang M., Xu Z., Yang L. (2021). miR-1301-3p Promotes Cell Proliferation and Facilitates Cell Cycle Progression via Targeting SIRT1 in Gastric Cancer. Front. Oncol..

[53] Peng Y., Feng H., Wang C., Song Z., Zhang Y., Liu K., Cheng X., Zhao R. (2021). The role of E26 transformation-specific variant transcription factor 5 in colorectal cancer cell proliferation and cell cycle progression. Cell Death Dis..

[54] Yang Q., Falahati A., Khosh A., Mohammed H., Kang W., Corachán A., Bariani M.V., Boyer T.G., Al-Hendy A. (2022). Targeting Class I Histone Deacetylases in Human Uterine Leiomyosarcoma. Cells.

[55] Liu Z., Duan Z., Zhang D., Xiao P., Zhang T., Xu H., Wang C.H., Rao G.W., Gan J., Huang Y. (2022). Structure–Activity Relationships and Antileukemia Effects of the Tricyclic Benzoic Acid FTO Inhibitors. J. Med. Chem..

[56] Hu F., Yan H.J., Gao C.X., Sun W.W., Long Y.S. (2023). Inhibition of Hypothalamic FTO Activates STAT3 Signal through ERK1/2 Associated with Reductions in Food Intake and Body Weight. Neuroendocrinology.

[57] Bushweller J.H. (2019). Targeting transcription factors in cancer from undruggable to reality. Nat. Rev. Cancer.

[58] Ala M. (2022). Target c-Myc to treat pancreatic cancer. Cancer Biol. Ther..

[59] Donati G., Amati B. (2022). MYC and therapy resistance in cancer: Risks and opportunities. Mol. Oncol..

[60] Fatma H., Maurya S.K., Siddique H.R. (2022). Epigenetic modifications of c-MYC: Role in cancer cell reprogramming, progression and chemoresistance. Semin. Cancer Biol..

[61] Roworth A.P., Ghari F., La Thangue N.B. (2014). To live or let die–complexity within the E2F1 pathway. Mol. Cell. Oncol..

[62] Fouad S., Hauton D., D’Angiolella V. (2021). E2F1: Cause and Consequence of DNA Replication Stress. Front. Mol. Biosci..

[63] Fang Z., Lin M., Li C., Liu H., Gong C. (2020). A comprehensive review of the roles of E2F1 in colon cancer. Am. J. Cancer Res..

[64] Huang Y., Chen R., Zhou J. (2016). E2F1 and NF-kappaB: Key Mediators of Inflammation-associated Cancers and Potential Therapeutic Targets. Curr. Cancer Drug Targets.

[65] Sun H., Ma H., Zhang H., Ji M. (2021). Up-regulation of MELK by E2F1 promotes the proliferation in cervical cancer cells. Int. J. Biol. Sci..

[66] Ke X.-Y., Chen Y., Tham V.Y., Lin R.Y., Dakle P., Nacro K., Puhaindran M.E., Houghton P., Pang A., Lee V.K. (2021). MNK1 and MNK2 enforce expression of E2F1, FOXM1, and WEE1 to drive soft tissue sarcoma. Oncogene.

[67] Tian S., Zhang L., Li Y., Cao D., Quan S., Guo Y., Yang X., Yang T. (2021). Human Papillomavirus E7 Oncoprotein Promotes Proliferation and Migration through the Transcription Factor E2F1 in Cervical Cancer Cells. Anti-Cancer Agents Med. Chem..

[68] Hemming M.L., Fan C., Raut C.P., Demetri G.D., Armstrong S.A., Sicinska E., George S. (2020). Oncogenic Gene-Expression Programs in Leiomyosarcoma and Characterization of Conventional, Inflammatory, and Uterogenic Subtypes. Mol. Cancer Res..

[69] Black A.R., Black J.D., Azizkhan-Clifford J. (2001). Sp1 and krüppel-like factor family of transcription factors in cell growth regulation and cancer. J. Cell. Physiol..

[70] Deng Y.R., Chen X.J., Chen W., Wu L.F., Jiang H.P., Kin D., Wang L.J., Wang W., Guo. S.Q. (2019). Sp1 contributes to radioresistance of cervical cancer through targeting G2/M cell cycle checkpoint CDK1. Cancer Manag. Res..

[71] Grinstein E., Jundt F., Weinert I., Wernet P., Royer H.-D. (2002). Sp1 as G1 cell cycle phase specific transcription factor in epithelial cells. Oncogene.

[72] Opitz O.G., Rustgi A.K. (2000). Interaction between Sp1 and cell cycle regulatory proteins is important in transactivation of a differentiation-related gene. Cancer Res..

[73] Bourassa M.W., Ratan R.R. (2014). The interplay between microRNAs and histone deacetylases in neurological diseases. Neurochem. Int..

[74] Baltan S., Sandau U.S., Brunet S., Bastian C., Tripathi A., Nguyen H., Liu H., Saugstad J.A., Zarnegarnia Y., Dutta R. (2021). Identification of miRNAs That Mediate Protective Functions of Anti-Cancer Drugs During White Matter Ischemic Injury. ASN Neuro.

[75] Klieser E., Urbas R., Swierczynski S., Stättner S., Primavesi F., Jäger T., Mayr C., Kiesslich T., Di Fazio P., Helm K. (2018). HDAC-Linked “Proliferative” miRNA Expression Pattern in Pancreatic Neuroendocrine Tumors. Int. J. Mol. Sci..

[76] Liu J., Dou X., Chen C.Y., Chen C., Liu C., Xu M.M., Zhao S., Shen B., Gao Y., Han D. (2020). *N*^6^-methyladenosine of chromosome-associated regulatory RNA regulates chromatin state and transcription. Science.

[77] Yang Q., Bariani M.V., Falahati A., Khosh A., Lastra R.R., Siblini H., Boyer T.G., Al-Hendy A. (2022). The Functional Role and Regulatory Mechanism of Bromodomain-Containing Protein 9 in Human Uterine Leiomyosarcoma. Cells.

[78] Ram S., Vizcarra P., Whalen P., Deng S., Painter C.L., Jackson-Fisher A., Pirie-Shepherd S., Xia X., Powell E.L. (2021). Pixelwise H-score: A novel digital image analysis-based metric to quantify membrane biomarker expression from immunohistochemistry images. PLoS ONE.

[79] Law C.W., Chen Y., Shi W., Smyth G.K. (2014). Voom: Precision weights unlock linear model analysis tools for RNA-seq read counts. Genome Biol..

[80] Kuleshov M.V., Jones M.R., Rouillard A.D., Fernandez N.F., Duan Q., Wang Z., Koplev S., Jenkins S.L., Jagodnik K.M., Lachmann A. (2016). Enrichr: A comprehensive gene set enrichment analysis web server 2016 update. Nucleic Acids Res..

