# Supplementary material for: The Functional Role and Regulatory Mechanism of FTO m6A RNA Demethylase in Human Uterine Leiomyosarcoma
Source: Int J Mol Sci. 2023 Apr 27;24(9):7957. doi: 10.3390/ijms24097957 (PMC10178470; doi:10.3390/ijms24097957)
Supplement: Supplementary file 1 [file ijms-24-07957-s001.zip › ijms-2234957-supplementary.pdf]

Figure S1. **Reactome analysis. (A) Upregulated DEGs; (B) Downregulated DEGs**

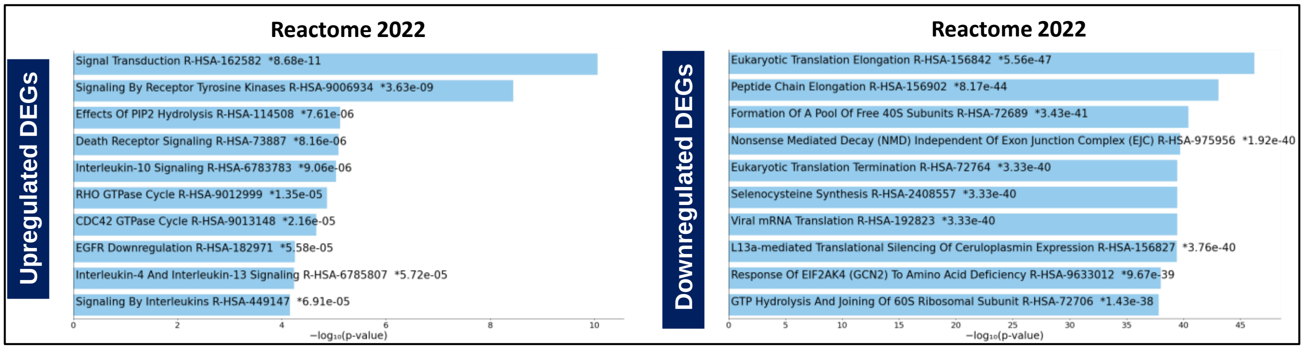

Figure S2. The expression of cell cycle-related genes and epigenetic regulators between DMSO and Dac51-treated LMS cells

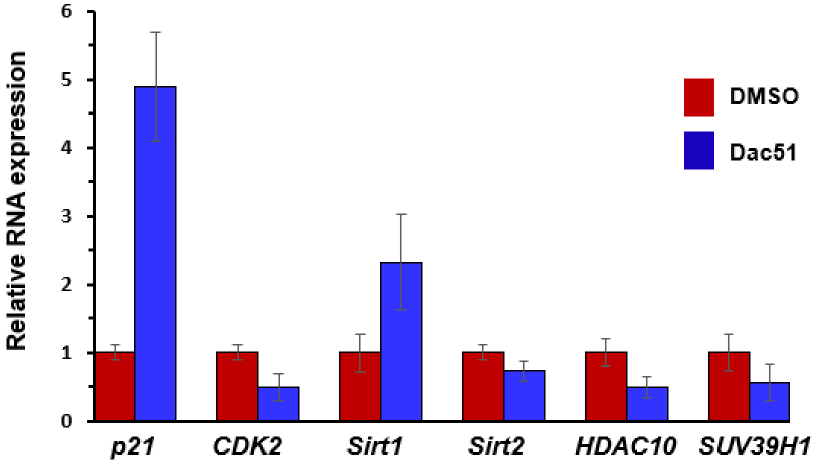

Table S1. Top 20 significant terms for ChEA of upregulated genes

Table S1. Top 20 significantly terms for ChEA of upregulated genes					
Term	Overlap	P-value	Adjusted P-value	Odds Ratio	Combined Score
NR3C1 27076634 ChIP-Seq BEAS2B Human Lung Inflammation	819/3084	2.30E-76	1.74E-73	2.466263743	429.5341622
SUZ12 20075857 ChIP-Seq MESCs Mouse	900/3611	6.86E-70	2.59E-67	2.281291823	363.3090392

ZNF217 24962896 ChIP-Seq MCF-7 Human	432/1289	1.10E-67	2.76E-65	3.194706569	492.5660972
NFKB1 27076634 ChIP-Seq BEAS2B Human Lung Inflammation	929/3823	1.46E-66	2.77E-64	2.20844174	334.775801
MBD3 35695185 ChIP-Seq nicBasalRootGanglia Mouse Embryo	881/3656	1.76E-60	2.67E-58	2.152228325	296.1200122
LEF1 29337183 ChIP-Seq mESC Mouse Stem	618/2335	1.32E-54	1.67E-52	2.329649164	289.0175064
TP63 23658742 ChIP-Seq EP156T Human	722/2937	3.15E-51	3.41E-49	2.13503386	248.2718351
AR 27270436 ChIP-Seq VCaP Human Prostate Carcinoma	830/3565	2.16E-49	2.04E-47	2.014178384	225.7050125
FOXA1 27270436 ChIP-Seq VCaP Human Prostate Carcinoma	768/3331	2.55E-43	2.15E-41	1.956379455	191.8689483
MTF2 20144788 ChIP-Seq MESCs Mouse	614/2488	3.49E-43	2.64E-41	2.095355361	204.8475143
WT1 25993318 ChIP-Seq PODOCYTE Human	689/2909	2.12E-42	1.46E-40	2.002927437	192.1994195
CJUN 26792858 Chip-Seq BT549 Human	430/1564	3.11E-41	1.96E-39	2.360122849	220.1322855
SMARCA4 23332759 ChIP-Seq OLIGODENDROCYTES Mouse	531/2089	1.17E-40	6.83E-39	2.149776178	197.6597557
SOX2 20726797 ChIP-Seq SW620 Human	499/1931	4.31E-40	2.33E-38	2.187335895	198.266821
CEBPD 21427703 ChIP-Seq 3T3-L1 Mouse	383/1349	6.02E-40	3.04E-38	2.448750395	221.1422026
TCF4 23295773 ChIP-Seq U87 Human	722/3177	5.30E-38	2.51E-36	1.895082458	162.6561887
CTCF 31629814 ChIP-Seq Hepatocytes Mouse Tyrosinemia	700/3086	2.20E-36	9.78E-35	1.881116328	154.4518938
STAT3 23295773 ChIP-Seq U87 Human	619/2637	2.65E-36	1.12E-34	1.947139309	159.503122
PPAR 26484153 Chip-Seq NCI-H1993 Human	287/945	2.26E-35	9.01E-34	2.647771681	211.2231764
MITF 21258399 ChIP-Seq MELANOMA Human	958/4614	4.00E-35	1.51E-33	1.729897142	137.0162036

Table S2. Top 20 significant terms for ChEA of downregulated genes

Term	Overlap	P-value	Adjusted P-value	Odds Ratio	Combined Score
MYC 18358816 ChIP-ChIP MESCs Mouse	497/2369	9.33E-30	7.07E-27	1.925931678	128.7367727
EGR1 20690147 ChIP-Seq ERYTHROLEUKEMIA Human	846/4931	7.37E-21	2.31E-18	1.539278579	71.35514076
E2F1 26619117 ChIP-Seq Hepatocytes Mouse Liver	517/2717	9.15E-21	2.31E-18	1.684425803	77.71990176
XRN2 22483619 ChIP-Seq HELA Human	286/1296	4.67E-20	8.84E-18	1.973491333	87.84054439

CCND1 20090754 ChIP-ChIP RETINA Mouse	314/1472	1.28E-19	1.93E-17	1.895293889	82.45616553
LXR 22292898 ChIP-Seq THP-1 Human	335/1614	5.71E-19	7.20E-17	1.833691601	77.02846218
E2F1 18555785 ChIP-Seq MESCs Mouse	551/3015	2.84E-18	3.08E-16	1.600677615	64.66913954
MYC 19030024 ChIP-ChIP MESCs Mouse	524/2842	4.17E-18	3.95E-16	1.613054042	64.55039866
UBF1/2 26484160 Chip-Seq HMEC-DERIVED Human	335/1650	1.88E-17	1.58E-15	1.779500773	68.53536042
FOXP1 21924763 ChIP-Seq HESCs Human	583/3273	6.12E-17	4.64E-15	1.55167123	57.92632242
SETDB1 19884255 ChIP-Seq MESCs Mouse	304/1478	1.16E-16	7.98E-15	1.800376411	66.06206696
MYBL2 22936984 ChIP-ChIP MESCs Mouse	293/1419	2.62E-16	1.66E-14	1.806034902	64.7943337
MYC 18555785 ChIP-Seq MESCs Mouse	196/852	7.29E-16	4.24E-14	2.048798002	71.41076091
PGR 26153859 ChIP-Seq MCF-7 Human BreastCancer	189/816	1.22E-15	6.59E-14	2.064659864	70.90334628
CEBPB 24764292 ChIP-Seq MC3T3 Mouse	296/1461	2.47E-15	1.25E-13	1.761471826	59.24529615
KLF4 18358816 ChIP-ChIP MESCs Mouse	257/1238	1.16E-14	5.39E-13	1.806719077	57.96753835
ZFP281 18757296 ChIP-ChIP E14 Mouse	294/1466	1.21E-14	5.39E-13	1.736882539	55.65740933
SOX9 25088423 ChIP-ChIP EMBRYONIC GONADS Mouse	317/1619	2.68E-14	1.13E-12	1.688847568	52.78019239
SA1 27219007 Chip-Seq ERYTHROID Human	321/1646	3.10E-14	1.21E-12	1.680969511	52.28412633
MYC 19079543 ChIP-ChIP MESCs Mouse	218/1013	3.20E-14	1.21E-12	1.881686003	58.47173526

Table S3. Top 20 significant terms for human microRNAs of upregulated genes

Term	Overlap	P-value	Adjusted P-value	Odds Ratio	Combined Score
hsa-miR-4704-5p	411/1869	5.41E-18	1.73E-16	1.706050231	67.83023287
hsa-miR-1244	389/1768	4.54E-17	1.03E-15	1.701342684	64.02204636
hsa-miR-21	412/1900	5.92E-17	1.16E-15	1.673141291	62.51669809
hsa-miR-590-5p	412/1900	5.92E-17	1.16E-15	1.673141291	62.51669809
hsa-miR-190	388/1779	2.19E-16	3.84E-15	1.680390571	60.58868405
hsa-miR-190b	388/1779	2.19E-16	3.84E-15	1.680390571	60.58868405
hsa-miR-3177-5p	400/1852	3.61E-16	6.01E-15	1.660787866	59.05345505
hsa-miR-331-5p	384/1775	1.21E-15	1.92E-14	1.660506423	57.03558631
hsa-miR-208a	382/1772	2.56E-15	3.82E-14	1.651880465	55.50016013
hsa-miR-208b	382/1772	2.56E-15	3.82E-14	1.651880465	55.50016013
hsa-miR-4671-3p	338/1528	3.27E-15	4.75E-14	1.700334355	56.71478181
hsa-miR-1284	409/1931	4.22E-15	5.89E-14	1.618409716	53.56653305
hsa-miR-4796-5p	349/1626	9.37E-14	9.87E-13	1.633864323	49.01417921
hsa-miR-4445	384/1826	1.02E-13	1.06E-12	1.596550852	47.7560986
hsa-miR-1278	361/1703	2.03E-13	1.96E-12	1.608877284	47.01751932
hsa-miR-3923	294/1335	4.54E-13	4.08E-12	1.678659342	47.70863868
hsa-miR-584	385/1855	6.75E-13	5.99E-12	1.567999707	43.94165927
hsa-miR-4764-3p	403/1960	7.34E-13	6.43E-12	1.551681819	43.35425769
hsa-miR-496	376/1812	1.36E-12	1.12E-11	1.565604978	42.78178607
hsa-miR-4703-3p	364/1749	2.18E-12	1.78E-11	1.569366204	42.13758089

Table S4. Top 20 significant terms for human microRNAs of downregulated genes

Term	Overlap	P-value	Adjusted P-value	Odds Ratio	Combined Score
hsa-miR-744	285/1290	4.62E-20	2.74E-17	1.976137202	87.9812621
hsa-miR-3180	310/1475	2.22E-18	3.03E-16	1.855902627	75.4441682
hsa-miR-3180-3p	310/1475	2.22E-18	3.03E-16	1.855902627	75.4441682
hsa-miR-3196	310/1475	2.22E-18	3.03E-16	1.855902627	75.4441682
hsa-miR-4467	189/803	2.22E-16	2.17E-14	2.110011489	76.05359407
hsa-miR-4706	254/1198	1.27E-15	8.50E-14	1.857461296	63.70584297
hsa-miR-4749-5p	254/1198	1.27E-15	8.50E-14	1.857461296	63.70584297
hsa-miR-1908	359/1849	1.37E-15	8.50E-14	1.682233064	57.5737493
hsa-miR-663	359/1849	1.37E-15	8.50E-14	1.682233064	57.5737493
hsa-miR-4532	319/1622	1.19E-14	6.75E-13	1.699560419	54.49828206
hsa-miR-4281	362/1918	7.17E-14	3.77E-12	1.619724615	49.02193599
hsa-miR-4508	232/1106	8.19E-14	3.99E-12	1.82340882	54.94562036
hsa-miR-4707-5p	232/1113	1.65E-13	7.04E-12	1.808153148	53.22074772
hsa-miR-4734	201/934	3.45E-13	1.37E-11	1.875535157	53.81960003
hsa-miR-4738-5p	193/887	3.61E-13	1.37E-11	1.900298224	54.44502528
hsa-miR-3141	189/879	1.98E-12	7.13E-11	1.869086672	50.36525419
hsa-miR-1538	213/1027	2.92E-12	9.50E-11	1.789849636	47.53769596
hsa-miR-4745-3p	213/1027	2.92E-12	9.50E-11	1.789849636	47.53769596
hsa-miR-4640-3p	332/1809	3.44E-11	1.07E-09	1.552261432	37.39888678
hsa-miR-1292	354/1959	5.15E-11	1.53E-09	1.525440327	36.13764101

Table S5. Primers used in the study

Gene symbol	Primer sequences	F or R	Assay	Species	Size (bp)	Accession
HDAC10	CCTGAGGGAGGAGACAGAA	F	q-PCR	Human	101	AF426160.1
HDAC10	TCCCATCTAAGAGGTACAGGAG	R	q-PCR	Human	101	
Sirt1	AGAACCCATGGAGGATGAAAG	F	q-PCR	Human	111	AF083106.2
Sirt1	TCATCTCCATCAGTCCCAAATC	R	q-PCR	Human	111	
Sirt2	GGACAACAGAGAGGGAGAAAC	F	q-PCR	Human	120	AY030277.1
Sirt2	AGACAAGAACTGCTGGTTAAGA	R	q-PCR	Human	120	
SUV39H1	CGAGGAGCTCACCTTTGATTAC	F	q-PCR	Human	122	NM_001282166.2
SUV39H1	CAATACGGACCCGCTTCTTAG	R	q-PCR	Human	122	
CDKN1A	CGGAACAAGGAGTCAGACATT	F	q-PCR	Human	105	NM_000389.5
CDKN1A	AGTGCCAGGAAAGACAACTAC	R	q-PCR	Human	105	
CDK2	AGATGGACGGAGCTTGTTATC	F	q-PCR	Human	103	X62071
CDK2	CTTGGTCACATCCTGGAAGAA	R	q-PCR	Human	103	
18S	CACGGACAGGATTGACAGATT	F	q-PCR	Human	119	NR_145820
18S	GCCAGAGTCTCGTTCGTTATC	R	q-PCR	Human	119	
